# Forecast of dementia prevalence in Germany and subnational regions until 2060 using microsimulation

**DOI:** 10.1007/s10654-026-01392-4

**Published:** 2026-05-04

**Authors:** Katrin Schüssel, Gabriela Brückner, Helmut Schröder, Gabriele Doblhammer, Frank Jessen, Julian Ernst, Christopher Caratiola, Ralf Münnich

**Affiliations:** 1 Research Institute of AOK: Wissenschaftliches Institut der AOK (WIdO), Berlin, Germany; 2 Chair of Empirical Methods in Social Science and Demography, Rostock University, Rostock, Germany; 3 Department of Psychiatry, Medical Faculty, University of Cologne, Cologne, Germany; 4 German Center for Neurodegenerative Diseases (DZNE), Bonn, Germany; 5 Economic and Social Statistics, Trier University, Trier, Germany

**Keywords:** Dementia, Prevalence, Microsimulation, Small area regional differences, Germany, Health claims data

## Abstract

The number of dementia cases is expected to rise in ageing societies with concomitant requirements for healthcare planning. While national figures for countries are widely available, subnational regional heterogeneity is rarely considered. This study aimed at projecting national and subnational dementia figures under different scenarios. Dementia and population figures where projected from 2018 to 2060 by microsimulation at the level of 400 counties within Germany. The German MikroSim model was supplemented with dementia-specific data on regional prevalence and national incidence and mortality figures. These data were derived from routine health insurance records of 27 million individuals within the AOK fund during pre-pandemic years 2017 to 2019 and adjusted to the total population of 2018. Scenarios with increasing life expectancy or prevention effects (decreasing dementia incidence by 1 to 2% per year) were calculated. Increasing life expectancy leads to dementia cases rising from 1.4 million in 2018 to 2.1 million in 2060, prevalence from 1.6 to 2.6% and the RWA (ratio of cases per 100 individuals of working age) from 2.6 to 4.7. In prevention scenarios, these figures are substantially lower: between 1.3 and 1.5 million cases, 1.6 and 1.9% prevalence and RWA between 2.9 and 3.4. Existing subnational regional differences in prevalence and RWA will become more pronounced in all scenarios. Growing life expectancy leads to an increase in dementia cases, which may be counteracted by preventive measures. Increasing regional heterogeneity in prevalence and RWA should be considered for health care planning.

**Supplementary Information:**

The online version contains supplementary material available at 10.1007/s10654-026-01392-4.

## Introduction

Dementia is one of the leading causes of disability in individuals of high age, affecting more than 20% of people aged 85 years and older in Western Europe [1–3]. The disease affects the well-being of patients, compromising orientation and communicative abilities, and patients frequently suffer psychiatric symptoms like anxiety, depression and sleep disturbance. Moreover, it often poses a strain on caring relatives [3]. Given global demographic trends with population growth and ageing, dementia cases are expected to increase more than 2.5-fold in the next decades [2]. Of course, this rise in case numbers is accompanied by growing societal costs, both direct costs for health care as well as even higher indirect costs for informal care [3–5].

Of note, the predicted trends vary for the different world regions. In regions like Western Europe, population growth is low. Therefore, dementia forecasts are mostly driven by population ageing, and the increase in case numbers is estimated to be less than twofold. Accordingly, at the national level of Germany, the relative increase in cases by 2050 is expected to be well below twofold: approximately 1.65- or 1.44-fold compared to 2019 or 2025, respectively [2, 3].

Given the rising numbers of individuals living with dementia in the future, health systems are required to adjust by increasing caregiving resources and develop preventive strategies [6, 7]. There may be substantial potential for prevention, as age-specific dementia incidence rates have decreased in high-income countries in recent decades [8, 9]. Although the cause for this decrease is not conclusively proven, it is often attributed to improvements in cardiovascular risk factors such as high blood pressure. A recent report based on a wide range of risk factors covering lifestyle, education, health-related and environmental factors estimated that up to 45% of dementia cases are potentially preventable [7]. Although this may represent an optimistic scenario and will likely not be achievable in the near future, the decreasing incidence trends nevertheless seem promising and could limit the number of future dementia cases.

There is a range of studies that predicted dementia case numbers on a global or national scale, all reporting a marked increase of dementia case numbers (cf [2, 10, 11]). On a national level for Germany, most studies are based on extrapolations combining age- and sex-specific dementia prevalence estimates with population forecasts from the national statistical office [3, 12, 13], while others employed multistate modelling [14, 15] or more complex model-based techniques [2]. All of these predictions might be adequate for generating national summaries, but it can be expected that dementia prevalence and also the demand for caregiving resources vary substantially by subnational regions. Hence, for more precise planning and allocation of future health care resources within countries, subnational estimates at the county level are required. So far, however, only one study in Germany produced subnational estimates at county level, utilizing an extrapolation approach, and projections were limited to the year 2030 [13]. Even international studies producing subnational estimates are scarce [16–18]. Therefore, the aim of this study was to generate forecasts of dementia prevalence up to the year 2060 both nationally and at subnational levels of 400 counties (NUTS-3) in Germany [19] using a microsimulation approach. Microsimulation methods have been employed in international studies for forecasts of dementia, frailty or caregiving needs in the elderly population, for example in Canada [20], England [21], Japan [22], the Netherlands [23, 24] or the US [25]. These studies produced estimates at the national level. In order to generate subnational estimates, the current study used a dynamic microsimulation approach integrating data on dementia-specific regional prevalence as well as national figures for incidence and mortality in Germany. Although this study focuses on Germany, the microsimulation methodology can be generalized to any country where subnational estimates of dementia prevalence and demographic figures are available.

## Methods

### Microsimulation with a high degree of regional resolution in Germany – the MikroSim model

Forecasts of dementia cases were generated by microsimulation within the MikroSim model version 2.1.6 [26]. The MikroSim model is a discrete-time spatial dynamic microsimulation, where simulation is based on yearly probabilities of an event in first-order Markov processes. The MikroSim model consists of several modules modelling demographic changes (mortality, ageing, births, regional mobility) followed by further modules for socioeconomic factors (partnership status, education, employment and income). Each module consists of one or more models estimated from data from the Federal Statistical Office of Germany (Destatis) or survey data like the German microcensus or the German Socio-Economic Panel. These source data are used to generate transition matrices containing probabilities for the event of interest (such as death, birth, regional mobility, etc.). For migration, official statistical data up to the year 2023 were used. Thus, up to 2023, the large migration effects from Ukraine are represented in the microsimulation results of this study [27]. Afterwards, future assumptions on migration were incorporated [28]. These assumptions are comparable to the moderate migration scenario of projections of the Federal Statistical Office of Germany [29].

For the prediction of regional dementia prevalence, the MikroSim data were augmented with dementia-specific figures for prevalence, incidence and mortality. In the first year of the microsimulation procedure, information on regional dementia prevalence by 400 counties in Germany by age and sex were used to simulate individuals having or not having dementia. For the death module of the MikroSim study, dementia excess mortality was used to model the death of individuals having dementia. New dementia cases were generated from transition probabilities for incidence. Overall, this procedure is equivalent to a 3-state illness-death-model where a healthy individual either dies (mortality of individuals without dementia) or gets the disease (incidence) and then dies after having had the disease (mortality of patients with dementia) [10].

### Dementia prevalence, mortality and incidence

Dementia-specific figures were generated from routine health insurance data of the AOK, which is the largest organization of health insurance providers in Germany, covering approximately 27 million individuals, i.e. one third of the total German population [30]. The case definition for dementia was based on inpatient and outpatient diagnoses for ICD-10-GM codes for Alzheimer’s disease (ICD F00, G30), vascular dementia (F01) and other forms of dementia or related disorders (F02, F03, F05.1, G23.1, G31.0, G31.82) utilizing an algorithm described previously [14]. For hospital cases, only discharge or secondary diagnoses were considered, while for outpatient cases, diagnoses had to be “verified” - a criterion specific to German health insurance data. All analyses were restricted to individuals with continuous health insurance histories of at least 10 years before the end of the database period or death, respectively, in order to ensure completeness of follow-up. For each case, periods with reversible dementia were excluded, since dementia was treated as an absorbing state, that is recovery from dementia is not modelled in the microsimulation approach, as described in the illness-death-model above. To this end, reversible dementia periods were defined as not having at least one repeat diagnosis within 3 consecutive quarters. If individuals died within 2 quarters from the dementia diagnosis, these periods were considered as dementia periods, even if no repeat diagnosis was made due to insufficient follow-up time. Applying these criteria, an individual may initially not be classified as having dementia during periods when the criterion for repeat diagnoses was not fulfilled (since the dementia diagnosis was not confirmed within 3 quarters). However, the same individual could later be classified as dementia once repeat diagnoses were recorded or death occured within 2 quarters after a dementia diagnosis. Overall, the number of dementia cases excluding reversible cases was approximately 15% lower than for all cases, which is in line with estimated frequencies of reversible dementia cases from the medical literature [31, 32] and with the proportion of persistent dementia versus all dementia cases reported by Zissimopoulos et al. [25].

Dementia prevalence based on data of AOK insurants was adjusted to the German population for age, sex and morbidity by regression-based modelling as described previously [33]. The adjustment method generates estimates by region (400 counties within Germany), sex and age groups. In order to exclude effects of the relatively short-term impact of the COVID-19 pandemic on dementia prevalence and/or mortality [34–36], average prevalence values were calculated from the pre-pandemic years 2017 to 2019. Projections based on the post-pandemic situation will only become possible in the near future (2026 and onwards), when the pandemic effects will have levelled off and appropriate post-pandemic health care data is available.

Incidence rates were generated from AOK-based health insurance data, again using average values from the years 2017 to 2019, i.e. before the COVID-19 pandemic. Incidence rates were calculated at the national level. For calculation of dementia incidence within the following year, only individuals without prior dementia were considered and followed for up to 4 quarters to detect incident dementia diagnoses. Dementia-free individuals dying during the follow-up-period were not considered for these calculations. This is consistent with the procedure in the MikroSim model, where dementia incidence calculation is performed after the mortality module, i.e. after individuals dying within the next year are removed from further consideration. AOK-specific dementia incidence rates were adjusted to the German population by deriving a factor for possible bias in morbidity in AOK patients relative to the total population. This bias-correcting factor was derived from the prevalence adjustment method [33] by 5-year age groups and sex at the national level. Thus, in each sex-specific age group the ratio of dementia prevalence in the population versus the prevalence for AOK-insured individuals was calculated and applied to the corresponding incidence rate. Consequently, if AOK prevalence in a specific age and sex group was higher compared to the prevalence in the total population, this relative difference was used to adjust AOK incidence rates downward accordingly to generate expected values in the total population.

For all-cause excess mortality, the probability of dying within the following year was calculated for AOK-insured individuals by 5-year age groups and sex, stratified by cases with and without dementia. As for incidence and prevalence, only data from pre-pandemic years 2017 to 2019 was utilized. This was deliberately chosen in order to obtain mortality rates for the long-term projections which are not distorted by higher values observed during the pandemic years [34–36]. Again, these calculations were performed at the national level. Mortality was calculated using AOK data according to standard demographic methods based on mortality tables [37]. Mortality of AOK insurees by age and sex was adjusted to official national mortality rates in the years 2018 to 2020 published by the Federal Statistical Office of Germany [37]. All-cause excess mortality of patients with dementia was computed as the mean of the mortality ratio and the mortality difference for individuals with dementia versus individuals without dementia, stratified by 5-year age groups and sex.

Figures for prevalence, excess mortality and incidence were each generated for 5-year age groups ranging from 40 to 120 years. To obtain smooth data by single years of age, the age-group-specific values were broken down into single-year values using B-spline functions. At the margin of the age distribution, specific assumptions were made for each epidemiology measure. Prevalence was assumed to be 0 for individuals under 40 years of age, while for individuals aged 94 years and older, a maximum age-specific prevalence of 55.1% for men and 60.2% for women was applied. Incidence at age 120 reached maximum values per 100 person-years of 12.8 for men and 12.3 for women. For mortality at age 120, the probability of dying within the following year cumulated at 92.1 per 100 person-years for both men and women without dementia and at 99.7 and 99.6 per 100 person-years for men or women with dementia, respectively. Figures for age- and sex-specific prevalence, incidence and mortality used in this study are provided in the supplementary material (Supplementary Figs. 1 to 3).

Mortality was analysed by dementia duration, defined as the number of years since the incident dementia diagnosis. However, the duration of dementia did not indicate an effect on mortality. A minor exception was slightly increased mortality among patients during their first year following the incident dementia diagnosis. However, this may rather reflect diagnostic bias, as patients with deteriorating clinical state in the year preceding their death may have sought medical advice more frequently, leading to a higher detection rate of cognitive decline and dementia diagnoses in routine health data [38]. Overall, the most relevant factors for mortality were age and sex. Therefore, the effect of the duration of dementia on mortality was not modelled in the microsimulation procedure.

### Baseline assumptions and scenarios for future mortality/life expectancy and dementia incidence

Several different scenarios were devised for this study (Table 1). In the first scenario S1, life expectancy is projected to increase in the future. This is considered the main scenario, as it can be assumed that the long-term trend of rising life expectancy observed in the years prior to the pandemic will continue in the coming years. The increase in life expectancy corresponds to a decrease in mortality, with yearly relative reductions of 0.85% for males and 0.75% for females. This scenario predicts a population figure of 79 million inhabitants in the year 2060 and aligns well with population projections from the Federal Statistical Office [29], estimating between 76 and 89 million inhabitants in the main scenarios, or between 76 and 85 million inhabitants in scenarios assuming low to moderate international migration to Germany. In further scenarios S2a to S2c, the increase in life expectancy is maintained, but it is accompanied by changes in dementia incidence with yearly relative reductions of 1.0, 1.4 or 2.0%, respectively. These assumptions are based on observations of an approximately 10% decline in dementia incidence per decade (corresponding to 1.4% per year) in international epidemiological studies [8, 9], as well as recent reports of reduced dementia incidence and prevalence in analyses from Germany based on routine health care data [39, 40] and outpatient diagnoses [41]. The reduction of dementia incidence is modelled for future years until a baseline value of 55% of the status-quo incidence is reached. This assumption is based on the premise that up to 45% of dementia cases could be preventable [7]. The incidence threshold of 55% is only relevant in scenario S2c with high incidence reduction of 2% per year, where the threshold is reached by the year 2048. For the other scenarios, the threshold limit is not reached by the year 2060. Finally, in a third scenario S3, both mortality/life expectancy as well as dementia incidence are assumed to remain at the status quo. This scenario represents a “pessimistic” scenario where rising trends in life expectancy from recent years are not extrapolated to the future.

**Table 1:** Scenarios for projections of future population and dementia cases

scenario	type	mortality assumption: relative reduction per year	Dementia incidence assumption: relative reduction per year (until threshold of 55% of status quo in 2018)
S1	increased life expectancy	0.85% for males 0.75% for females	unchanged - status quo (2018)
S2a	increased life expectancy PLUS reduced dementia incidence		1.0%
S2b			1.4%
S2c			2.0%
S3	status quo	unchanged - status quo (2018)	unchanged - status quo (2018)

### Sensitivity analysis including reversible dementia cases and dementia recovery as fourth transition state

In addition to the above-mentioned 3-state illness-death-model, a sensitivity analysis was performed including all dementia cases and adding a fourth transition state of recovery from dementia to the illness-death model. Probabilities of recovery from dementia within the following year were generated analogous to incidence rates: at a national level, derived from AOK-based health insurance data, using average values from the years 2017 to 2019 and smoothing of data by single years of age using B-spline functions.

### Software

All statistical calculations were performed with R [42]. The versions of R, R Studio IDE (Posit Software) and R packages employed are listed in the supplementary material.

### Reporting

Reporting of this study, where applicable, follows recommendations of the RECORD statement (REporting of studies Conducted using Observational Routinely collected health Data) [43].

## Results

### Absolute numbers of population and dementia cases

**Fig. 1:**
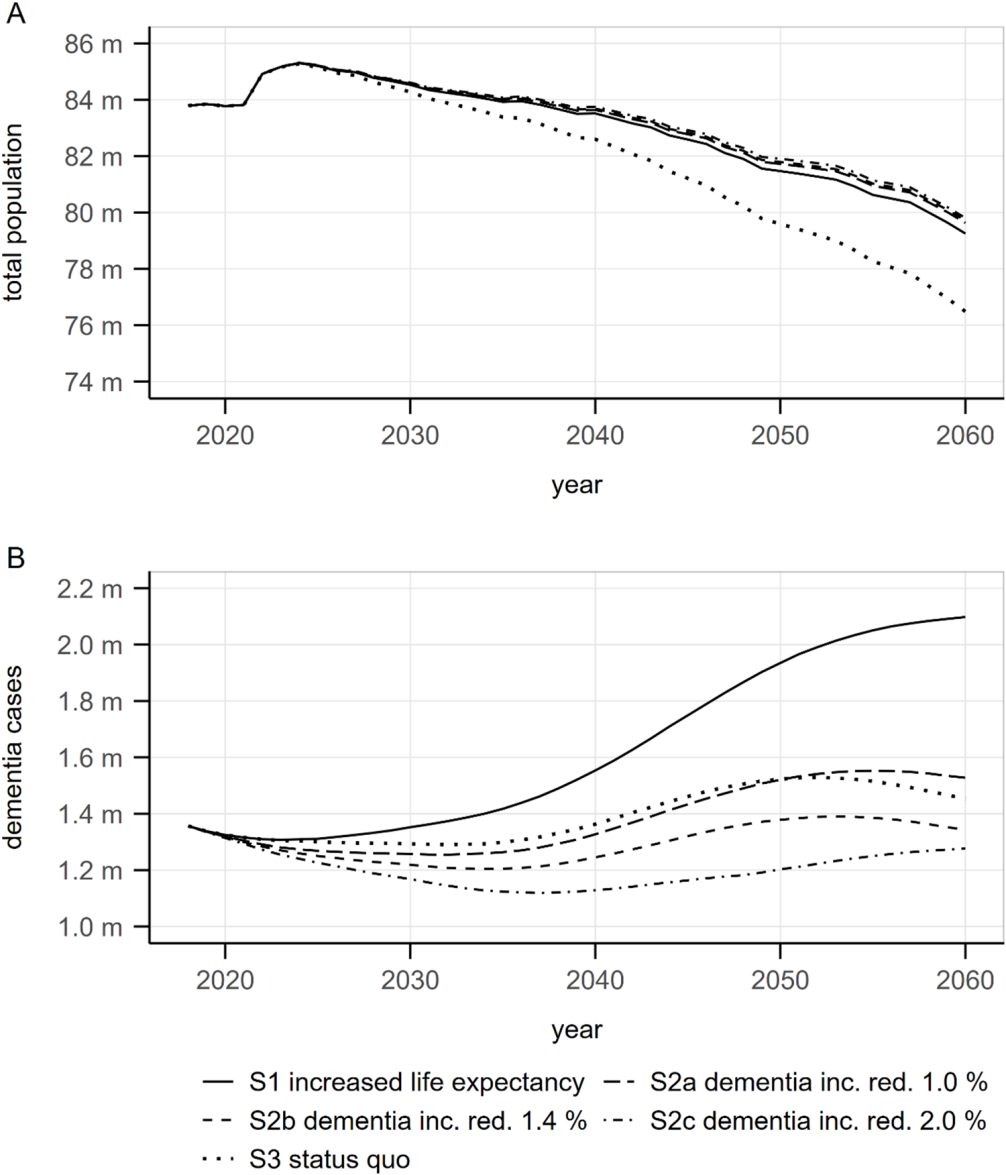
Projections of total population figures (upper panel) and dementia cases (lower panel) up to the year 2060 in the main scenario S1 (increasing life expectancy and constant dementia incidence, solid line), in prevention scenarios S2a to S2c (increasing life expectancy and decreasing dementia incidence with dashed, short-dashed and dotted-dashed lines for small, medium and high reduction of dementia incidence) and in the “status quo” scenario S3 (constant life expectancy and constant dementia incidence, dotted line)

In scenarios with increasing life expectancy (main scenario S1 as well as prevention scenarios S2a to S2c), projected population figures result in approximately 79 million inhabitants in 2060 (Fig. 1, upper panel, solid, dashed and dotted-dashed lines). In these scenarios, assumptions of decreasing dementia incidence in scenarios S2a to S2c have only a modest effect on population figures, resulting in slightly higher numbers in scenarios with decreased dementia incidence. In contrast, population figures are much lower in the “status quo” scenario S3 where life expectancy and dementia incidence remain unchanged, with only 76.5 million inhabitants in the year 2060 (Fig. 1, upper panel, dotted line).

The number of dementia cases shows a small reduction in most scenarios until the late 2030 s, followed by an increase afterwards. The highest number of dementia cases is projected in the main scenario S1 with increasing life expectancy and unchanged dementia incidence, reaching a peak of 2.1 million cases in 2060 (Fig. 1, lower panel, solid line). This is approximately 1.5-fold the level in the starting year 2018. In prevention scenarios S2a to S2c with decreasing dementia incidence, case numbers are considerably lower, with the lowest value of 1.28 million cases in 2060 under the assumption of a high reduction in dementia incidence (Fig. 1, lower panel, dotted-dashed line). In the ”status quo” scenario S3 with unchanged life expectancy and dementia incidence, dementia figures rise until the early 2050 s (Fig. 1, lower panel, dotted line), corresponding to the large cohort of “baby boomers” born in Germany in the mid-1950s/1960s having reached an age of 85 years and older. (The population pyramid displaying the age structure of the Germany population in 2018 is provided in the supplementary material, see Supplementary Fig. 4).

The results of sensitivity analyses including reversible dementia cases and modelling recovery from dementia as an additional fourth transition state in the illness-death model resulted in higher case numbers of dementia and prevalence (see Supplementary Figs. 5 and 6), but the patterns of the time course and effects of the different scenarios are comparable to the main analysis. The results of the main analyses can therefore be considered a conservative estimate of future dementia numbers.

### Dementia prevalence in the total population and ratio to the population of working age (RWA)

**Fig. 2:**
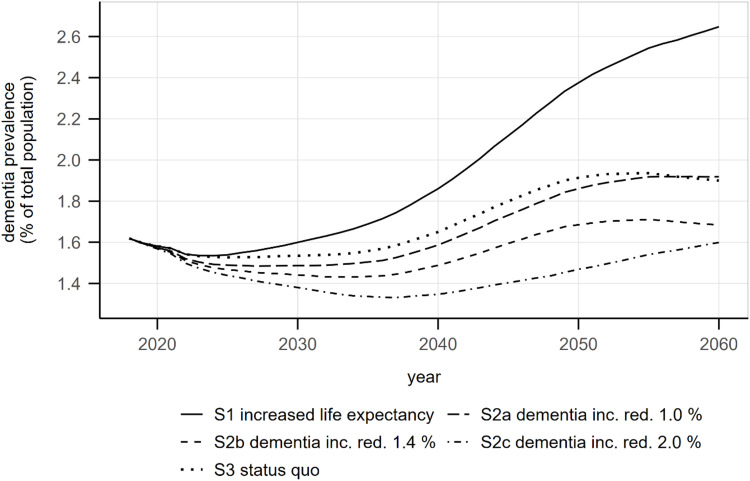
Projections of dementia prevalence up to the year 2060 in the main scenario S1 (increasing life expectancy and constant dementia incidence, solid line), in prevention scenarios S2a to S2c (increasing life expectancy and decreasing dementia incidence with dashed, short-dashed and dotted-dashed lines for small, medium and high reduction of dementia incidence, respectively) and in the “status quo” scenario S3 (constant life expectancy and constant dementia incidence, dotted line)

Corresponding to the absolute population and dementia figures, relative dementia prevalence initially declines before increasing in the 2030 years (Fig. 2). As can be expected, increasing life expectancy in combination with unchanged dementia incidence in the main scenario S1 leads to a substantial rise in dementia prevalence, cumulating at more than 2.6% in 2060 (Fig. 2, solid line). In scenarios with decreasing dementia incidence, prevalence is considerably lower, ranging from 1.6 to 1.9% in 2060 (dashed, short-dashed and dotted-dashed lines).

**Fig. 3:**
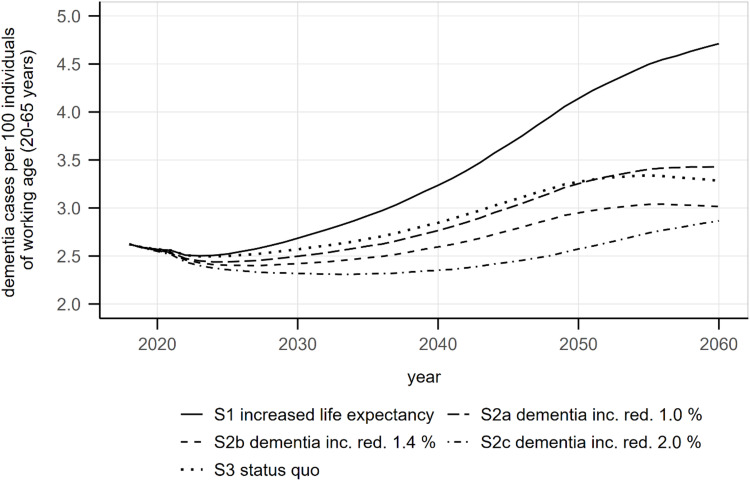
Ratio of dementia cases per working-age population (RWA) in Germany up to the year 2060 in the main scenario S1 (increasing life expectancy and constant dementia incidence, solid line), in prevention scenarios S2a to S2c (increasing life expectancy and decreasing dementia incidence with dashed, short-dashed and dotted-dashed lines for small, medium and high reduction of dementia incidence) and in the “status quo” scenario S3 (constant life expectancy and constant dementia incidence, dotted line)

In addition to prevalence, the ratio of dementia cases versus the population of working age (RWA) may be of interest for allocation of health care resources. For these analyses, inhabitants between 20 and 65 years of age were considered as working population. In the main scenario S1 with increasing life expectancy, the RWA rises markedly from 2.6 to 4.7 dementia cases per 100 working-age individuals by 2060 (Fig. 3, solid line). In the other scenarios, the RWA is lower, but still increased over the baseline RWA of 2.6 in 2018. Even in the best-case scenario with strongly reduced dementia incidence, RWA is predicted at 2.9 cases per 100 working-age individuals in the year 2060 (Fig. 3, dotted-dashed line).

### Dementia prevalence by age

**Fig. 4:**
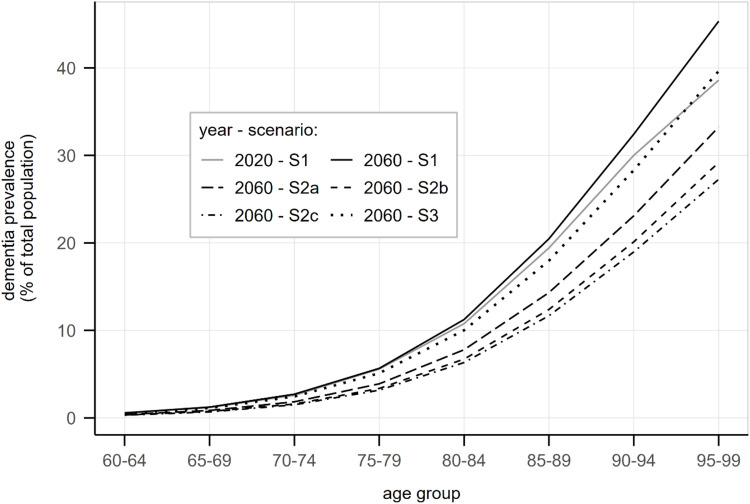
Projections of dementia prevalence for different years and scenarios. Main scenario S1 with increasing life expectancy: year 2020 grey solid line, year 2060 black solid line. Prevention scenarios S2a to S2c with increasing life expectancy and decreasing dementia incidence in year 2060: dashed, short-dashed and dotted-dashed lines for small, medium and high reduction of dementia incidence, respectively. “Status quo” scenario S3 in year 2060: dotted line

Dementia prevalence by age increases in older age groups in the main scenario S1 with rising life expectancy (Fig. 4, solid lines). In this scenario, prevalence in the year 2060 peaks at 45% in the age group of 95 to 99 years. In scenarios S2a to S2c with decreasing dementia incidence, prevalence is considerably lower, between 27 and 33% in this age group in 2060. In the “status quo” scenario S3, dementia prevalence in the year 2060 remains stable at comparable levels to the year 2020 (Fig. 4, dotted line). Results on national dementia prevalence by age and sex under all scenarios for the decades from 2020 to 2060 are available as csv-file in the supplementary material.

### Dementia prevalence and ratio of dementia cases per working-age population (RWA) by subnational regions

**Fig. 5:**
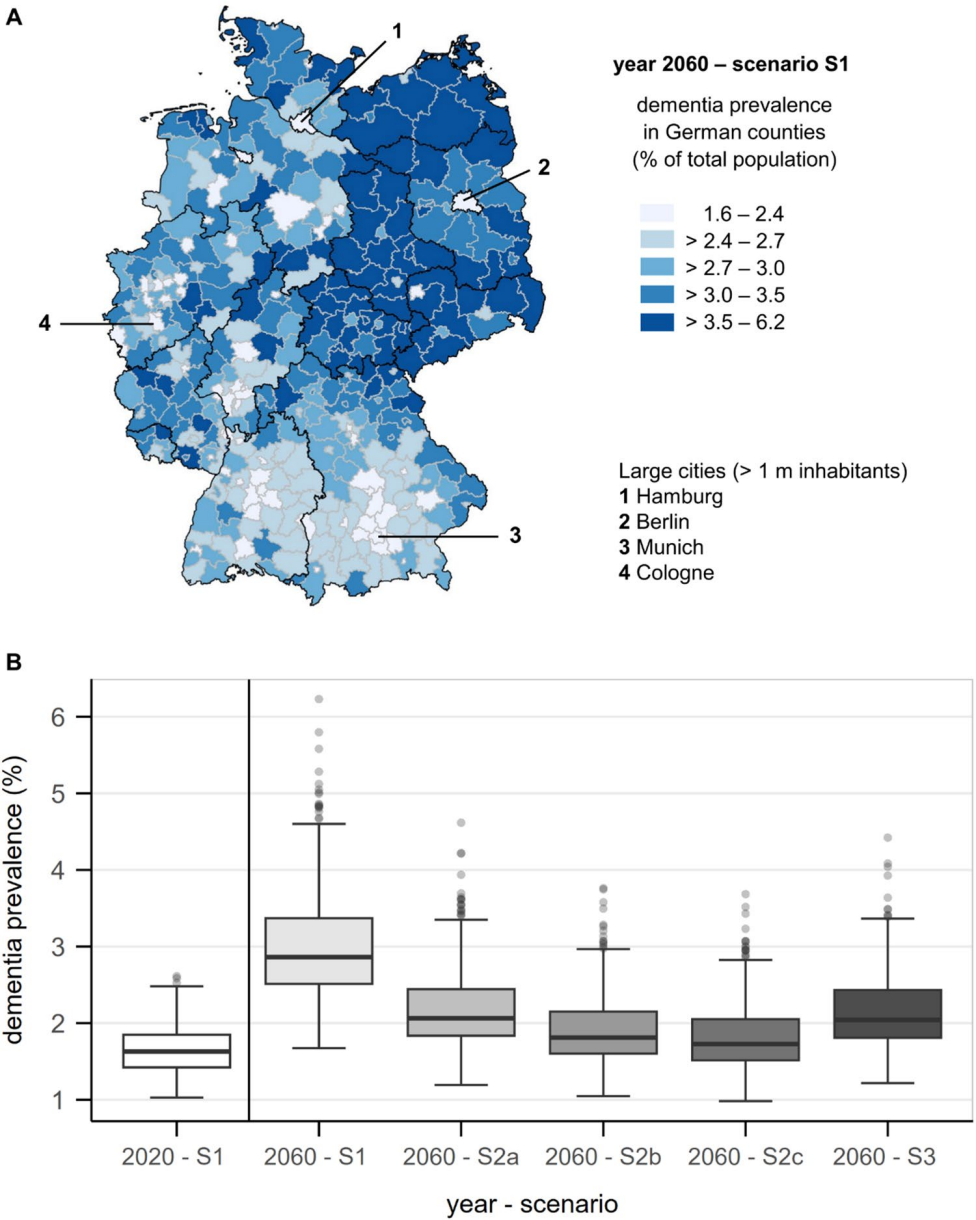
Regional projections of dementia prevalence for 400 counties in Germany in the years 2020 (main scenario S1) and 2060 (all scenarios). Upper panel **A**: cloropleth map of dementia prevalence in 400 German counties for scenario S1 in the year 2060. Lower panel **B**: box plot of dementia prevalence in 400 German counties in the year 2020 (main scenario S1) and 2060 (all scenarios: main scenario S1 with increasing life expectancy, prevention scenarios S2a/S2b/S2c with low/medium/high reduction of dementia incidence, “status quo” scenario S3)

**Fig. 6:**
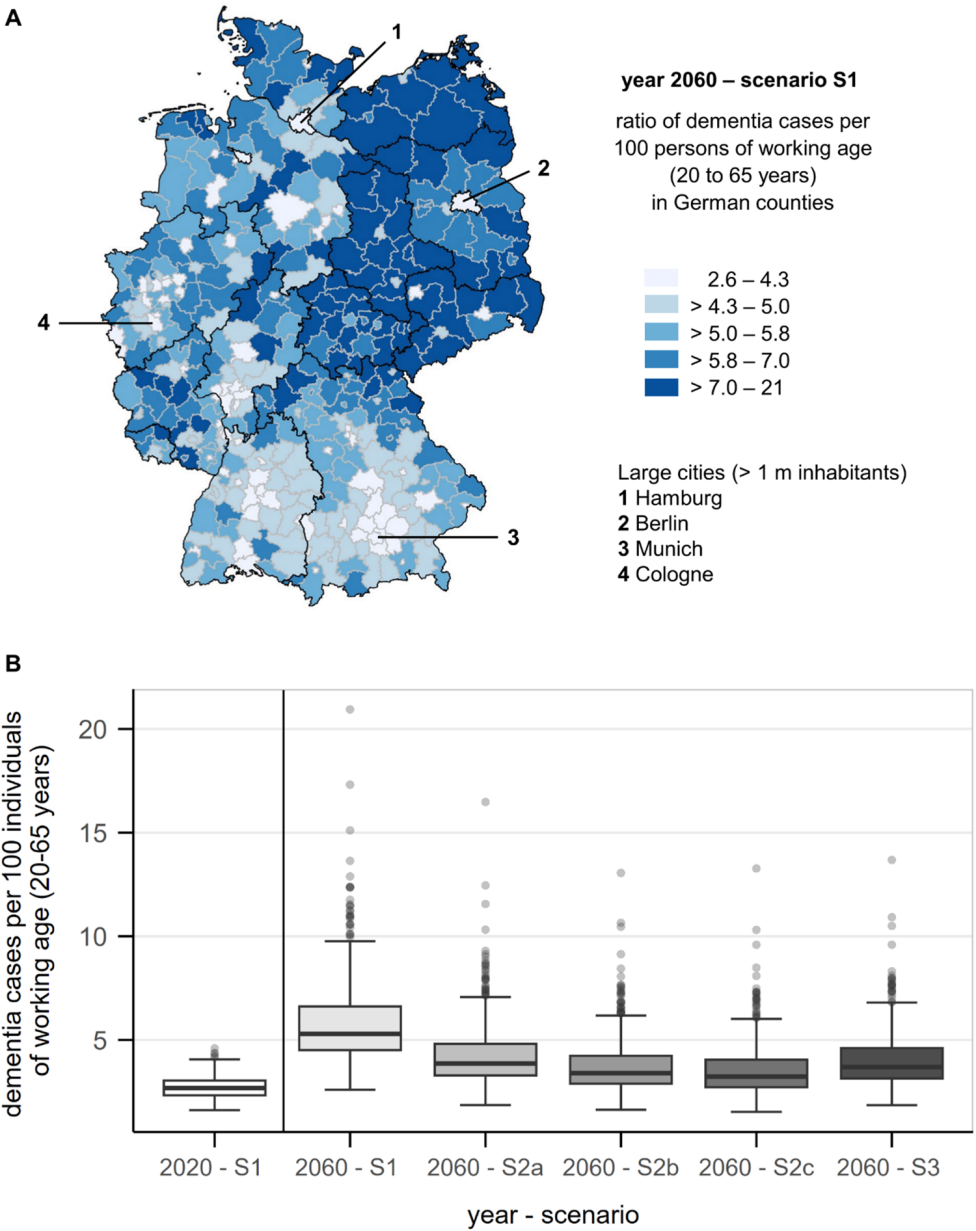
Regional projections of the ratio of dementia cases to working-age population (RWA) for 400 counties in Germany in the years 2020 (main scenario S1) and 2060 (all scenarios). Upper panel **A**: cloropleth map of RWA in 400 German counties for scenario S1 in the year 2060. Lower panel **B**: box plot of RWA in 400 German counties in the year 2020 (main scenario S1) and 2060 (all scenarios: main scenario S1 with increasing life expectancy, prevention scenarios S2a/S2b/S2c with low/medium/high reduction of dementia incidence, “status quo” scenario S3)

The microsimulation method generates projections of dementia prevalence and RWA across 400 subnational regions (counties) in Germany (Figs. 5 and 6, respectively). In the main scenario S1 with increasing life expectancy, high dementia prevalence of up to 6% by 2060 is projected in some eastern German counties (Fig. 5A, upper panel map). Large cities such as Berlin, Hamburg, Munich and Cologne have both low dementia prevalence and a low RWA (upper panel maps in Figs. 5A and 6A, respectively). An analysis of regional heterogeneity among the 400 counties in Germany in the year 2020 shows only relatively modest differences in the S1 scenario (Figs. 5B and 6B, left boxes): Dementia prevalence ranges from 1.0 to 2.6% with an interquartile range of 0.4 (Fig. 5B box plot, left box) and RWA from 1.6 to 4.6 with an interquartile range of 0.7 (Fig. 6B box plot, left box). However, by the year 2060, existing subnational differences in dementia prevalence and RWA across the 400 counties in Germany are amplified in all scenarios (Figs. 5B and 6B, boxes for the year 2060 on the right). Results on regional dementia prevalence and RWA in all scenarios for the decades from 2020 to 2060 are available as csv-file in the supplementary material.

As expected based on the methodology used, this heterogeneity is due to differences in regional population projections. Accordingly, the age- and gender-standardized values for the prevalence of dementia and the RWA in the 400 counties are very similar and show only minor deviations due to chance effects (results not shown).

## Discussion

The results of this study provide projections of dementia cases in Germany over the next decades under varying assumptions: while increasing life expectancy will result in higher case numbers, this effect is alleviated in prevention scenarios. The microsimulation projections allow for a high degree of regional granularity. Through this we were able to reveal heterogeneity among subnational regions (counties) not only in dementia prevalence but also in the ratio of dementia cases to the population of working age.

### Projections of the number of dementia cases

First of all, assuming rising life expectancy in the main scenario S1, a substantial increase in dementia figures is expected by 2060, rising by a factor of 1.5 from 1.4 million patients in 2018 to more than 2.1 million in 2060. This finding is generally comparable with other projections of dementia cases in Germany. For example, the projections by Georges et al. predict a 1.44- to 1.55-fold relative increase in dementia cases by 2050 compared 2025 [3], and GBD projections exhibit a 1.65-fold increase by 2050 relative to 2019 [2]. Our results reinforce previous analyses that the increase in life expectancy is a major contributor to rising dementia cases, both in studies from Germany [3, 12–15] as well as internationally [2, 10, 20–22, 24, 25]. This increase in dementia cases is generally consistent irrespective of the methodology used – whether extrapolations [12, 13], macrosimulations with illness-death-models [10, 14, 15], microsimulations [20–22, 24, 25] or complex regression-based modelling [2]. Our current microsimulation results align well with these previous studies.

However, the absolute number of dementia cases may differ between studies, depending on case definition and prevalence data used. In the current study, the case definition was based on routine health insurance data and reversible cases of dementia were excluded, resulting in a relative loss of approximately 15% of total dementia cases. This proportion lies within the range of expected values [25, 31, 32]. Thus, the number of dementia cases in Germany in 2018 was estimated at approximately 1.4 million patients, slightly lower than estimates from other studies, which range from 1.5 to 1.8 million patients in Germany in the years 2015 to 2025 [2, 3, 12–15]. In the sensitivity analyses including reversible dementia cases, the number of dementia cases was 1.6 million patients in 2018. The results of the main analyses (excluding reversible cases) may therefore be considered a rather conservative estimate of future case numbers of dementia.

In the current study, we observed a reduction in dementia prevalence in the starting years of the microsimulation until the 2030 s, even in the status quo scenario. A possible explanation is an imbalance between the starting year prevalence and incidence, resulting in decreasing prevalence in the following years if incidence is relatively low. This concept of low incidence aligns with observations of decreasing time trends in dementia incidence that have been observed in recent years – both in analyses based on routine healthcare data in Germany [39, 41] as well as in international epidemiologic studies [8, 9]. Hence, the 2018 dementia prevalence in the starting year of the current microsimulation reflects patients whose dementia state began years before, i.e. when incidence rates were still higher. Combining these (relatively high) prevalence data from 2018 with the (lower) incidence data from the same year will effectively result in microsimulation projections of lower dementia prevalence in the following years. Of note, this result of declining dementia prevalence projections until the mid 2020 s is consistent with decreasing dementia prevalence observed in analyses of German routine healthcare data in recent years [40, 41].

### Effects of prevention scenarios

Regardless of the case definition for dementia, numerous studies established that the future number of dementia cases is greatly reduced in preventive scenarios, i.e. when dementia incidence decreases [3, 12, 14, 15, 20, 24]. This effect was also observed in the current microsimulation study, in which dementia prevalence in an ageing population can be stabilized or even reduced in the most optimistic scenario. In this scenario, age- and sex-specific dementia prevalence shows a marked reduction in older age groups by the year 2060. Although there is evidence for a reduction in dementia incidence in recent years from European and US studies [8, 9] and also specifically from German studies [39–41], the scenario assuming a 2% yearly reduction in incidence may be overly optimistic. In the more realistic middle prevention scenario assuming a 1.4% yearly reduction, the number of dementia cases in 2060 stabilizes at approximately 1.4 million cases – similar to the number of dementia cases in the microsimulation starting year 2018. Nevertheless, it remains uncertain whether the observed decline in dementia incidence will continue in coming years, and whether the full potential of up to 45% preventable dementia cases can be exhausted [7], given that trends for some risk factors, such as obesity, may only stabilize rather than improve in the future [44, 45].

### Detailed results from microsimulation: subnational estimates and analyses in relation to the working-age population

An advantage of the current microsimulation approach in dementia projections is the high degree of details in the results. This allows, for example, to produce subnational estimates for dementia prevalence. There are no comparable studies, only one extrapolation of dementia prevalence using population estimates from the national statistical office for the year 2030 [13]. In this study, regional patterns are roughly similar to the current results, with dementia prevalence being higher in several rural regions of Eastern Germany, where the share of elderly people in the population is higher.

Another example to illustrate the degree of detail in the microsimulation results is the opportunity to contrast dementia cases with different population subgroups, such as the population of working age. Such analyses are highly relevant for planning the provision of long-term care for the elderly. The results indicate that the proportion of individuals with dementia relative to the population of working age (RWA) will almost double from 2.6 dementia cases per 100 individuals of working age in 2018 to 4.7 in 2060 in the scenario assuming increasing life expectancy. Thus, the increase in dementia cases contrasts with a reduction in the population of working age. Even in prevention scenarios, where reduced dementia incidence stabilizes the number of dementia cases, the RWA rises to values between 2.9 and 3.4. This would still be a considerable increase over the RWA of 2.6 in the year 2018. These figures highlight that even under the most optimistic scenario, society has to face a rising demand for care provision for individuals with dementia. This is consistent with findings from other microsimulation studies in Canada, England and Japan, which predict an increase in the number of individuals requiring care and associated societal costs [20–22].

Moreover, the simulation results indicate that existing subnational heterogeneity in dementia prevalence and RWA population will become more pronounced in all scenarios. This is presumably due to the combined effects of population ageing and migration, as younger people are more likely to move [46]. Therefore, regions facing an ageing population as well as a drain of young inhabitants, which are often rural regions in Eastern Germany [47], will exhibit both higher dementia prevalence and a higher RWA. Existing regional differences in these figures may therefore be aggravated in the future.

### Limitations

The current study underlies some limitations, which arise from the source data on dementia prevalence, incidence and mortality as well as from assumptions underlying future scenarios.

The case definition of dementia was based on medical diagnoses recorded in routine health insurance data. Therefore, dementia prevalence and incidence figures may differ from population-based epidemiologic studies utilizing cognitive test performance to establish dementia cases. However, previous studies based on German routine healthcare data employing a comparable case definition have shown that dementia prevalence estimates are broadly comparable to results from epidemiologic studies [39, 48]. Secondly, cases of reversible dementia were excluded in the current study, resulting in slightly lower dementia figures compared to other studies, as discussed above. Thirdly, incidence rates were also derived from routine health insurance data using the same case definition excluding reversible dementia cases. This may explain why incidence rates in this study are slightly lower than those reported by other studies from Germany including reversible cases [3]. In contrast, incidence rates in the current study were slightly higher compared to those used in Canadian and US microsimulation studies [20, 25]. However, the comparability of prevalence and incidence rates with international studies is limited due to methodological differences in case definitions. Moreover, excess mortality of individuals with dementia was calculated from routine health insurance data and may also deviate from results from other studies. However, one study from Germany reported mortality rates within 1 year at age 80 for individuals without and with dementia, which were 0.04 and 0.14 for men, and 0.02 and 0.10 for women, respectively [14]. The corresponding values used in the current study are quite comparable with 0.043 and 0.161 for men, and 0.026 and 0.120 for women. Furthermore, when comparing mortality rates in terms of relative values, the general observation that dementia excess mortality is more pronounced in young individuals and levels off in higher age matches well with other studies [20, 49]. Also, the relative mortality risk for individuals with dementia compared to those without dementia between ages 65 and 94 was on average 1.6 for men and 1.7 for women, which lies within the (wide) range of 1.4 to 5.6 in the review of epidemiologic studies by Todd et al. [49]. Despite these possible limitations of the source data, the microsimulation results are generally comparable with other studies. Therefore, the data on dementia incidence and excess mortality used in this study can be considered plausible.

Apart from limitations of the source data, the projection scenarios inherently depend on assumptions about future demographics changes including birth rates, life expectance/mortality and migration. For the scenario with increased life expectancy, the resulting population projections align well with the medium scenario of population forecasts from the Federal Statistical Office of Germany [29], indicating that the underlying assumptions are generally plausible. However, official projections at the county (NUTS-3 [19]) level are not available and thus could not be used for more detailed comparisons. Additionally, sub-national differences in life expectancy, dementia incidence and dementia excess mortality were not taken into account due to lack of data. Future projections may consider regional differences in these parameters, if valid data sources allow their estimation e.g. by utilizing small area methods [50, 51]. Moreover, the projections start in 2018, i.e. before the COVID-19 pandemic, and do not incorporate putative short- or long-term effects of the pandemic.

The projections of dementia cases in our scenarios were greatly influenced by assumptions regarding dementia incidence. In the prevention scenarios, it was assumed that dementia incidence would decline by relative values of 1, 1.4 or 2% per year, until reaching a threshold of 55% of the dementia incidence observed in the source data (i.e. the status quo in the year 2018). This assumption was based on reports of reduced dementia incidence and prevalence [8, 9, 39], and the 55% threshold value was chosen based on the estimated preventability of up to 45% of dementia cases [7]. These assumptions strongly affect the projected number of future cases and the scenario assuming a high reduction in dementia incidence may be overly optimistic.

Finally, the current microsimulation did not model the effects of specific risk factors, due to lack of data on their prevalence and association with dementia in Germany. However, microsimulation studies from other countries with available epidemiologic data have included dementia-specific risk factors [21–23, 25]. For future research, it would be desirable that epidemiologic studies in Germany provide detailed data on dementia and exposure to risk factors, including their effects across the lifespan [7, 52, 53]. This would enable incorporation of these risk factors in future microsimulations. Notably, many risk factors for dementia, such as hypertension, do not only affect dementia risk, but also life expectancy. Therefore, improvements in these risk factors may lower dementia incidence but could be offset by increasing longevity, resulting in more dementia cases at older ages. Depending on assumptions and modelling strategies in dementia projections, studies integrating such risk factors reported either a pronounced reduction [54], a slight reduction [23] or unchanged estimates of dementia prevalence [10], or even an increased lifetime risk for dementia and years lived with dementia [25]. Nevertheless, previous studies have shown that dementia prevalence would be greatly reduced by including factors that specifically delay dementia onset in the modelling approach, i.e. preventive treatments [10, 25]. However, judging from the development in the last 20 years, such preventive dementia treatments are unlikely to be available in the foreseeable future.

## Conclusion

This study demonstrates the impact of varying scenarios on future dementia cases and highlights the demand for planning care resources at both a national and subnational level. Accordingly, these projections are relevant for policy makers and healthcare providers. The regional microsimulation approach offers a high degree of details, enabling analyses of regional differences in dementia prevalence across subnational units and contrasting the number of dementia cases with different population subgroups such as the population of working age. Moreover, the methodology can be adapted to include additional variables such as dementia-specific risk factors or educational attainment, dependency on long-term care as well as subnational differences in life expectancy. Given the availability of such data, future projections should aim to expand the variables and effects included in the microsimulation models.

## Supplementary Information

Below is the link to the electronic supplementary material.

Supplementary Material 1 Additional methodology and results information (figures for the year 2018 on prevalence, incidence, mortality and population pyramid, results of the sensitivity analysis including reversible cases as well as detailed information on R software versions and packages).

Supplementary Material 2 Csv file with results for national dementia prevalence by age and sex (for all scenarios per decades from 2020 to 2060).

Supplementary Material 3 Csv file with results on regional dementia prevalence and RWA (for all scenarios per decades from 2020 to 2060).

## Data Availability

Pseudonymized billing data from the Scientific Institute of the AOK (WIdO) was used for the study. This data set cannot be made publicly available. Requests for aggregate data on which the results are based can be sent by email to the corresponding author. Raw data and programming code is not publicly available, but may be provided for non-commercial use upon request.
