# Supplementary material for: Forecast of dementia prevalence in Germany and subnational regions until 2060 using microsimulation
Source: Eur J Epidemiol. 2026 May 4;41(5):651–64. doi: 10.1007/s10654-026-01392-4 (PMC13332974; doi:10.1007/s10654-026-01392-4)
Supplement: Supplementary file 1 — Supplementary Material 1 Additional methodology and results information (figures for the year 2018 on prevalence, incidence, mortality and population pyramid, results of the sensitivity analysis including reversible cases as well as detailed information on R software versions and packages). [file 10654_2026_1392_MOESM1_ESM.docx]

# **Supplementary file**

**Forecast of dementia prevalence in Germany and subnational regions until 2060 using microsimulation**

**Katrin Schüssel^1^, Gabriela Brückner^1^, Helmut Schröder^1^, Gabriele Doblhammer^2^, Frank Jessen^3,4^, Julian Ernst^5^, Christopher Caratiola^5^, Ralf Münnich^5^**

**1 Research Institute of AOK: Wissenschaftliches Institut der AOK (WIdO), Berlin, Germany**

**2 Chair of Empirical Methods in Social Science and Demography, Rostock University, Rostock, Germany**

**3 Department of Psychiatry, Medical Faculty, University of Cologne, Cologne, Germany**

**4 German Center for Neurodegenerative Diseases (DZNE), Bonn, Germany**

**5 Trier University, Economic and Social Statistics, Trier, Germany**

**Corresponding author: Katrin Schüssel,** [katrin.schuessel@wido.bv.aok.de](mailto:katrin.schuessel@wido.bv.aok.de)

ORCIDs:

**Katrin Schüssel 0000-0002-8377-2254 https://orcid.org/0000-0002-8377-2254**

**Gabriela Brückner 0009-0000-4464-5351 https://orcid.org/0009-0000-4464-5351**

**Helmut Schröder 0009-0008-1447-6794 https://orcid.org/0009-0008-1447-6794**

**Gabriele Doblhammer 0000-0001-7746-0652 https://orcid.org/0000-0001-7746-0652**

**Frank Jessen** **0000-0003-1067-2102 https://orcid.org/0000-0003-1067-2102**

**Julian Ernst** **0009-0008-3679-1488 https://orcid.org/0009-0008-3679-1488**

**Christopher Caratiola 0009-0008-3548-9367 https://orcid.org/0009-0008-3548-9367**

**Ralf Münnich 0000-0001-8285-5667 https://orcid.org/0000-0001-8285-5667**

# Dementia prevalence by age and sex

*
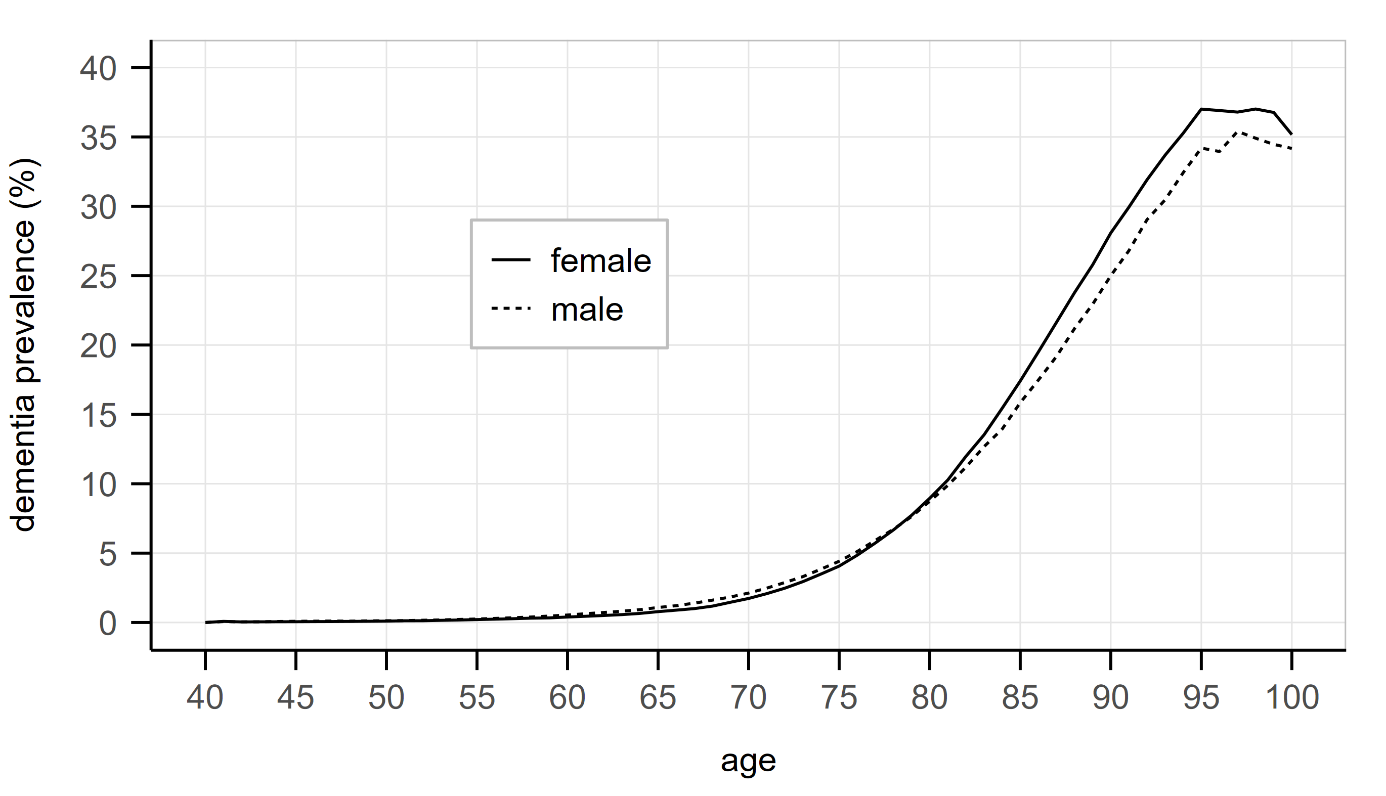
*

***Supplementary Figure S1*** *dementia prevalence (percent) by age and sex
(females: solid line, males: dotted line)*

# Dementia incidence by age and sex


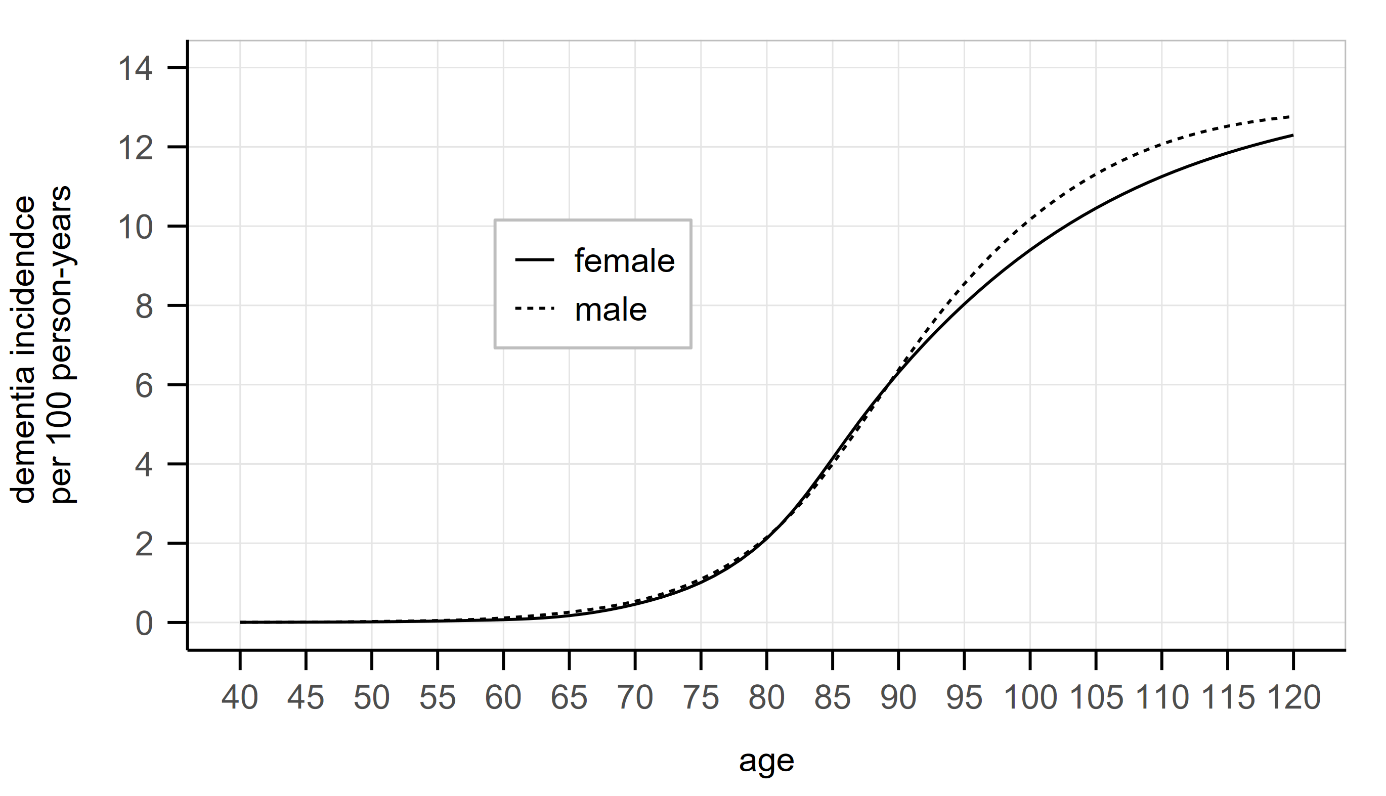


***Supplementary Figure S2*** *dementia incidence per 100 person-years by age and sex
(females: solid line, males: dotted line)*

# Mortality by age, sex and dementia status


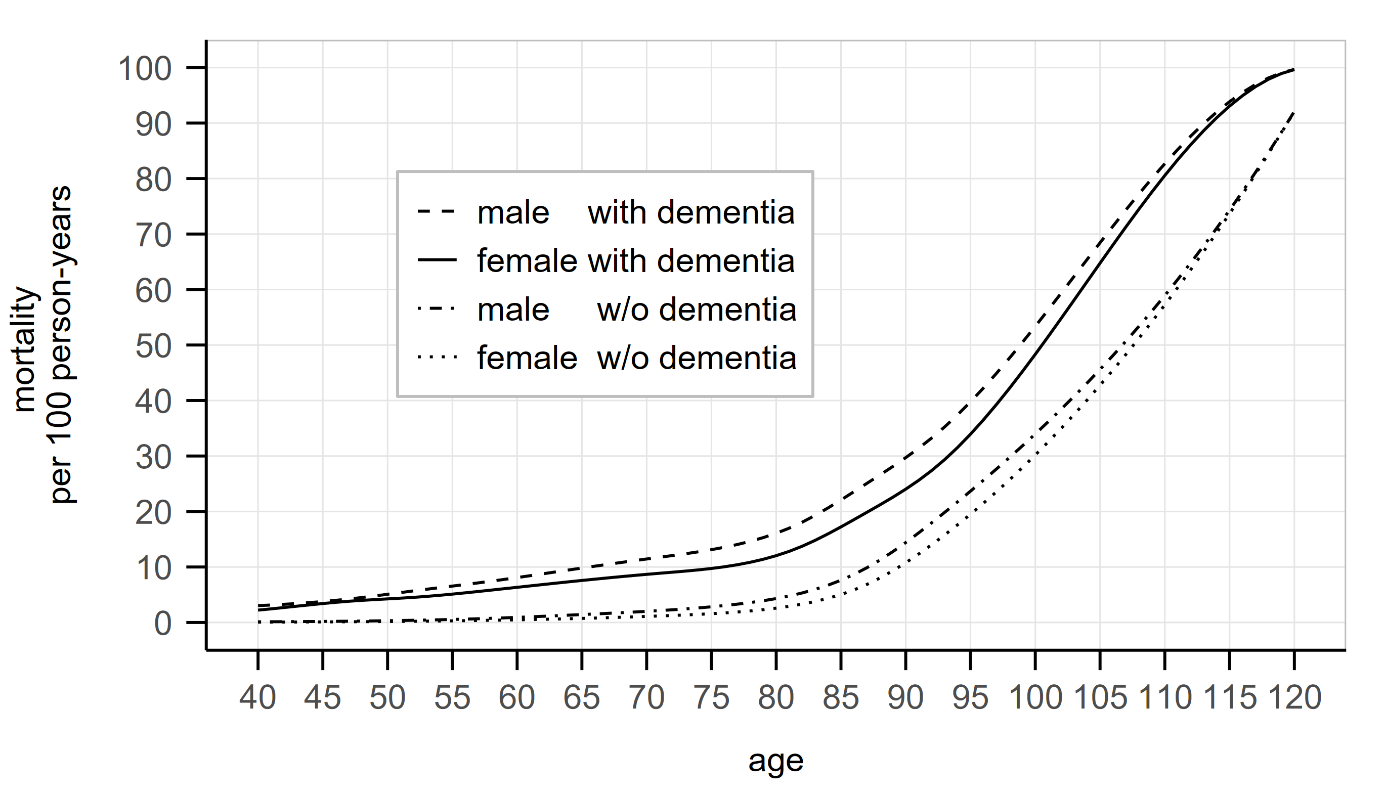


***Supplementary Figure S3*** *mortality per 100 person-years by age, sex and dementia status
(males with dementia: dashed line, females with dementia: solid line,
males without dementia: dotted-dashed line, females without dementia: dotted line)*

# Population pyramid for Germany in 2018


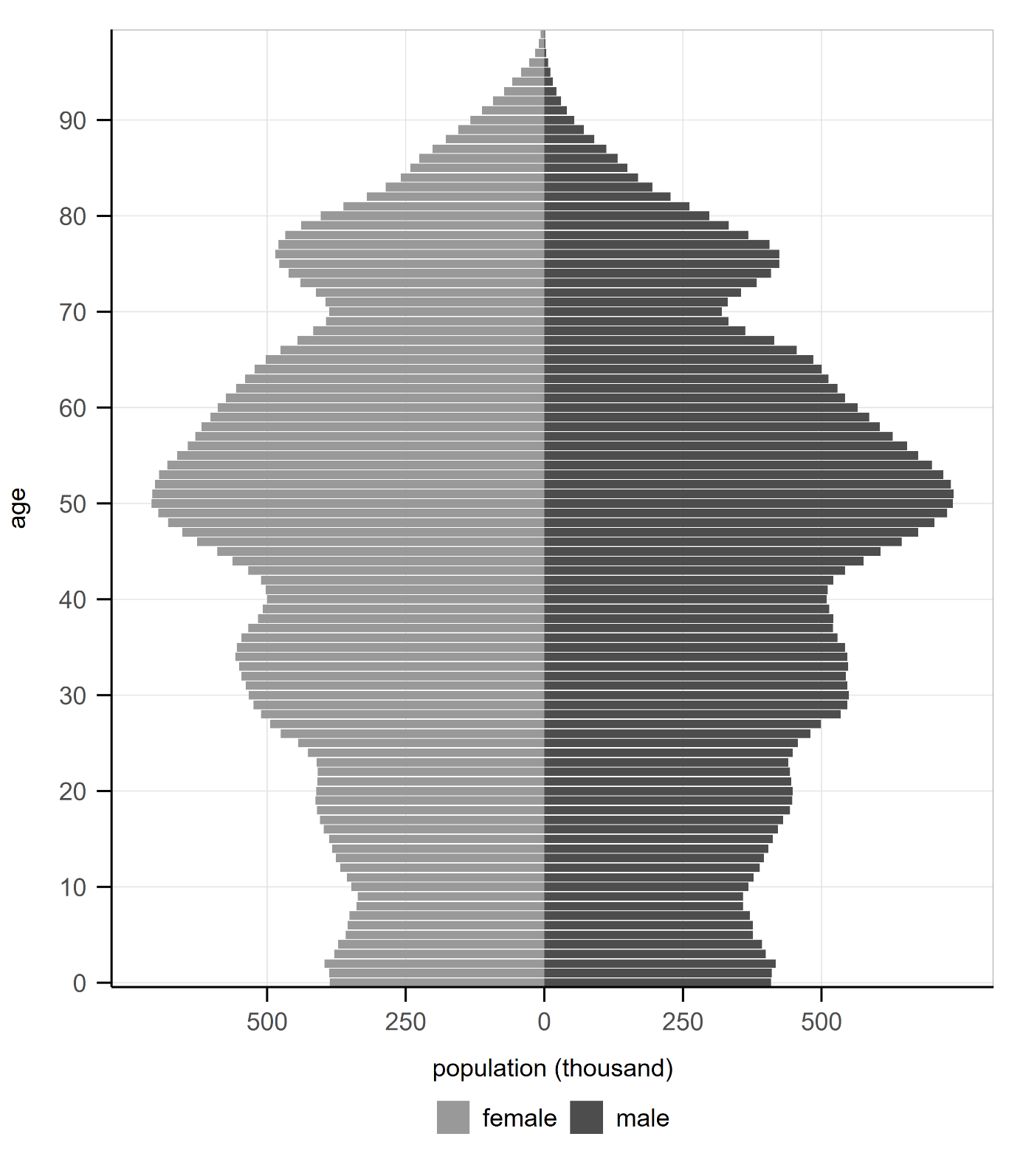


***Supplementary Figure S4*** *population pyramid
(numbers of population by age and sex, grey = females, dark grey = males).*

# Sensitivity analysis including reversible cases: number of dementia cases


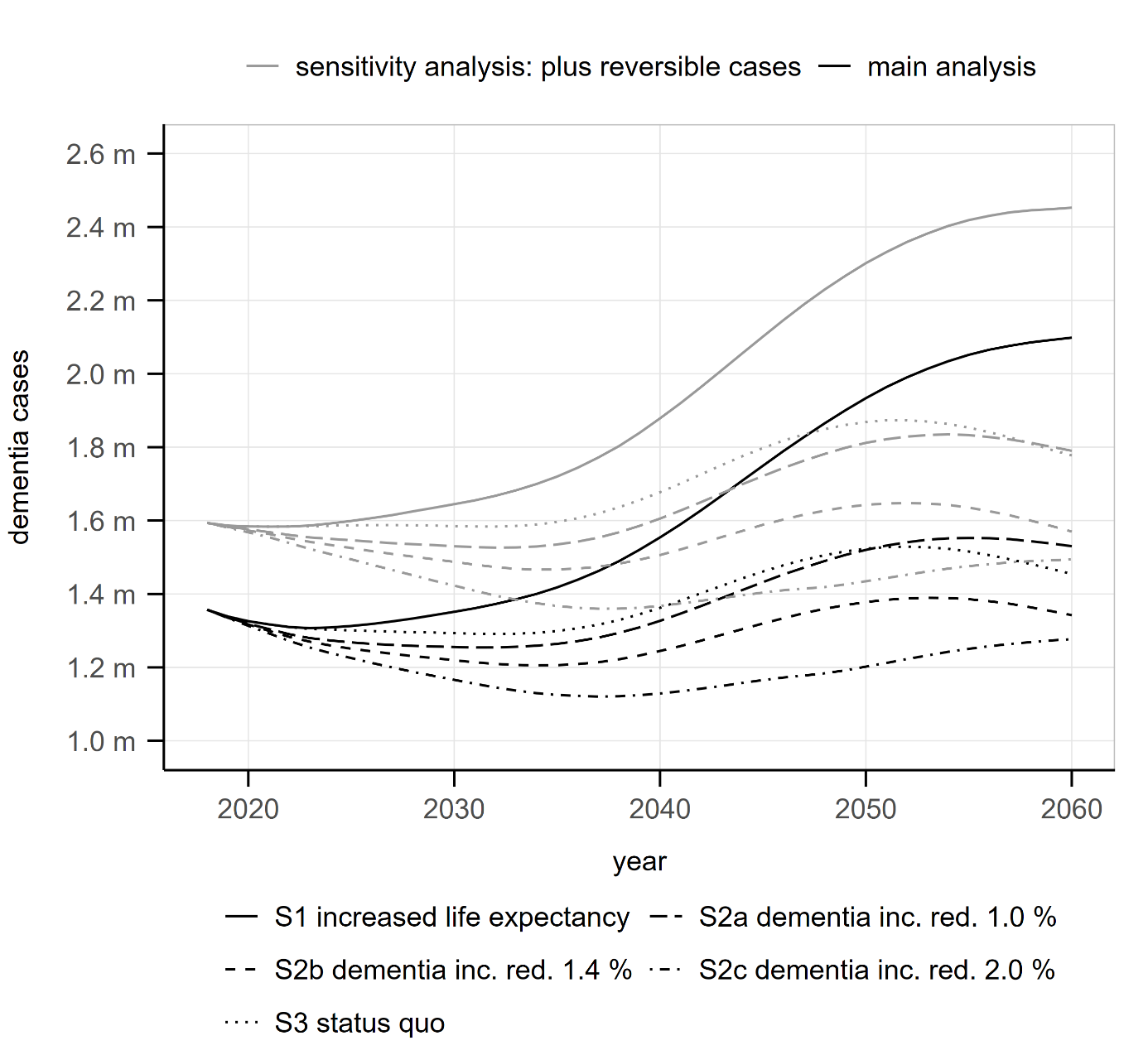


***Supplementary Figure S5*** *Projections of dementia cases up to the year 2060 in main analysis (black lines) and the sensitivity analysis including reversible cases (grey lines) for all scenarios: scenario S1 (increasing life expectancy and constant dementia incidence, solid line), in prevention scenarios S2a to S2c (increasing life expectancy and decreasing dementia incidence with dashed, short-dashed and dotted-dashed lines for small, medium and high reduction of dementia incidence, respectively) and in the “status quo” scenario S3 (constant life expectancy and constant dementia incidence, dotted line).*

# Sensitivity analysis including reversible cases: dementia prevalence


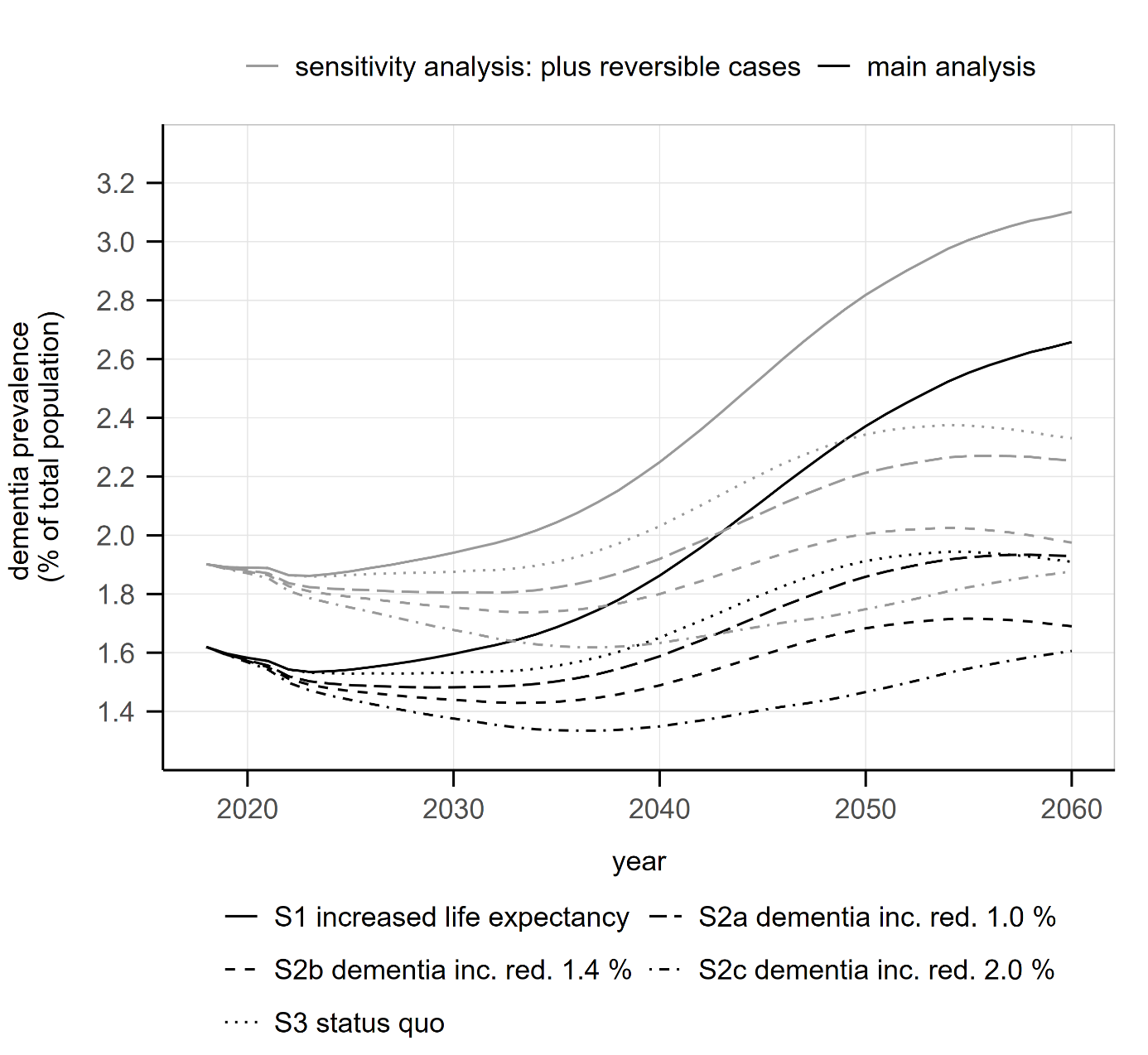


***Supplementary Figure S6*** *Projections of dementia prevalence up to the year 2060 in main analysis (black lines) and the sensitivity analysis including reversible cases (grey lines) for all scenarios: scenario S1 (increasing life expectancy and constant dementia incidence, solid line), in prevention scenarios S2a to S2c (increasing life expectancy and decreasing dementia incidence with dashed, short-dashed and dotted-dashed lines for small, medium and high reduction of dementia incidence, respectively) and in the “status quo” scenario S3 (constant life expectancy and constant dementia incidence, dotted line).*

# Software

All statistical calculations were performed with R in different versions and using different packages as detailed below.

## Preparation of dementia prevalence, incidence and mortality data

Calculations of dementia prevalence, incidence and mortality were performed with R version 4.2.2 utilizing R Studio (Posit Software: RStudio 2024.04.0+735 "Chocolate Cosmos" Release (a00d0e77, 2024-04-24) for windows. <https://posit.co/products/open-source/rstudio/>). The packages employed are listed in supplementary table 1.

**Supplementary table 1:** R packages employed for preparation of dementia prevalence, incidence and mortality data

| **package** | **version** | **citation** |
| --- | --- | --- |
|  | 4.2.2 | R Core Team (2022). R: A language and environment for statistical computing. R Foundation for Statistical Computing, Vienna, Austria.  https://www.R-project.org/ |
| boot | 1.3-28.1 | Canty A, Ripley BD (2021). boot: Bootstrap R (S-Plus) Functions.  Davison AC, Hinkley DV (1997). Bootstrap Methods and Their Applications. Cambridge University Press, Cambridge. ISBN 0-521-57391-2. |
| cAIC4 | 1.0 | Säfken B, Rügamer D, Kneib T, Greven S (2021). Conditional Model Selection in Mixed-Effects Models with cAIC4. Journal of Statistical Software, 99(8), 1-30. DOI 10.18637/jss.v099.i08 |
| data.table | 1.14.2 | Dowle M, Srinivasan A (2021). data.table: Extension of `data.frame`. |
| DBI | 1.2.3 | R Special Interest Group on Databases (R-SIG-DB), Wickham H, Müller K (2024). DBI: R Database Interface. |
| doParallel | 1.0.16 | Corporation M, Weston S (2020). doParallel: Foreach Parallel Adaptor for the 'parallel' Package. |
| dplyr | 1.0.7 | Wickham H, François R, Henry L, Müller K (2021). dplyr: A Grammar of Data Manipulation. |
| forcats | 0.5.1 | Wickham H (2021). forcats: Tools for Working with Categorical Variables (Factors). |
| foreach | 1.5.2 | Microsoft, Weston S (2022). foreach: Provides Foreach Looping Construct. |
| ggplot2 | 3.4.2 | H. Wickham. ggplot2: Elegant Graphics for Data Analysis. Springer-Verlag New York, 2016. |
| ggpubr | 0.4.0 | Kassambara A (2020). ggpubr: 'ggplot2' Based Publication Ready Plots. |
| gridExtra | 2.3 | Auguie B (2017). gridExtra: Miscellaneous Functions for "Grid" Graphics. |
| iterators | 1.0.14 | Analytics R, Weston S (2022). iterators: Provides Iterator Construct. |
| lattice | 0.20-45 | Sarkar, Deepayan (2008) Lattice: Multivariate Data Visualization with R. Springer, New York. ISBN 978-0-387-75968-5 |
| lme4 | 1.1-34 | Bates D, Mächler M, Bolker B, Walker S (2015). Fitting Linear Mixed-Effects Models Using lme4. Journal of Statistical Software 67(1), 1-48. DOI 10.18637/jss.v067.i01 |
| Matrix | 1.5-4 | Bates D, Maechler M, Jagan M (2023). Matrix: Sparse and Dense Matrix Classes and Methods. |
| mgcv | 1.8-41 | Wood SN (2011). Fast stable restricted maximum likelihood and marginal likelihood estimation of semiparametric generalized linear models. Journal of the Royal Statistical Society (B) 73(1), 3-36.  Wood S , Pya N, Säfken B (2016). Smoothing parameter and model selection for general smooth models (with discussion). Journal of the American Statistical Association 111, 1548-1575.  Wood SN (2004). Stable and efficient multiple smoothing parameter estimation for generalized additive models. Journal of the American Statistical Association 99(467), 673-686.  Wood S (2017). Generalized Additive Models: An Introduction with R, 2nd edition. Chapman and Hall/CRC.  Wood SN (2003). Thin-plate regression splines. Journal of the Royal Statistical Society (B), 65(1), 95-114. |
| mice | 3.13.0 | Stef van Buuren, Karin Groothuis-Oudshoorn (2011). mice: Multivariate Imputation by Chained Equations in R. Journal of Statistical Software, 45(3), 1-67. https://www.jstatsoft.org/v45/i03/. |
| nlme | 3.1-162 | Pinheiro J, Bates D, R Core Team (2023). nlme: Linear and Nonlinear Mixed Effects Models.  Pinheiro JC, Bates DM (2000). Mixed-Effects Models in S and S-PLUS. Springer, New York. DOI 10.1007/b98882 |
| odbc | 1.3.2 | Hester J, Wickham H (2021). odbc: Connect to ODBC Compatible Databases (using the DBI Interface). |
| purrr | 0.3.4 | Henry L, Wickham H (2020). purrr: Functional Programming Tools. |
| readr | 2.1.0 | Wickham H, Hester J (2021). readr: Read Rectangular Text Data. |
| readxl | 1.3.1 | Wickham H, Bryan J (2019). readxl: Read Excel Files. |
| RODBC | 1.3-19 | Ripley B, Lapsley M (2021). RODBC: ODBC Database Access. |
| stringr | 1.5.0 | Wickham H (2022). stringr: Simple, Consistent Wrappers for Common String Operations. |
| tibble | 3.1.4 | Müller K, Wickham H (2021)._tibble: Simple Data Frames. |
| tidyr | 1.1.4 | Wickham H (2021)._tidyr: Tidy Messy Data. |
| tidyverse | 1.3.1 | Wickham et al., (2019). Welcome to the tidyverse. Journal of Open Source Software, 4(43), 1686. DOI 10.21105/joss.01686 |
| varhandle | 2.0.5 | Mahmoudian M (2020). varhandle: Functions for Robust Variable Handling |
| writexl | 1.5.2 | Ooms J (2025). writexl: Export Data Frames to Excel 'xlsx' Format. |

## Microsimulation

For microsimulation, version 3.6.3 was used with the packages listed in supplementary table 2.

**Supplementary table 2:** R packages employed for microsimulation

| **package** | **version** | **citation** |
| --- | --- | --- |
|  | 3.6.3 | R Core Team (2022). R: A language and environment for statistical computing. R Foundation for Statistical Computing, Vienna, Austria.  https://www.R-project.org/ |
| collapse | 2.0.19 | Krantz, S. (2024). collapse: Advanced and Fast Statistical Computing and Data Transformation in R [Preprint]. arXiv. https://arxiv.org/abs/2403.05038  Krantz (2025). collapse: Advanced and Fast Data Transformation in R. R package version 2.1.3. doi:10.5281/zenodo.8433090. https://sebkrantz.github.io/collapse/. |
| dplyr | 1.0.7 | Wickham H, François R, Henry L, Müller K (2021). dplyr: A Grammar of Data Manipulation. |
| data.table | 1.14.2 | Dowle M, Srinivasan A (2021). data.table: Extension of `data.frame`. |
| mgcv | 1.8.-38 | Wood SN (2011). Fast stable restricted maximum likelihood and marginal likelihood estimation of semiparametric generalized linear models. Journal of the Royal Statistical Society (B) 73(1), 3-36.  Wood S , Pya N, Säfken B (2016). Smoothing parameter and model selection for general smooth models (with discussion). Journal of the American Statistical Association 111, 1548-1575.  Wood SN (2004). Stable and efficient multiple smoothing parameter estimation for generalized additive models. Journal of the American Statistical Association 99(467), 673-686.  Wood S (2017). Generalized Additive Models: An Introduction with R, 2nd edition. Chapman and Hall/CRC.  Wood SN (2003). Thin-plate regression splines. Journal of the Royal Statistical Society (B), 65(1), 95-114. |
| VGAM | 1.1-5 | Yee TW (2015). Vector Generalized Linear and Additive Models: With an Implementation in R. Springer, New York, USA.  Yee TW, Wild CJ (1996). “Vector Generalized Additive Models.” Journal of Royal Statistical Society, Series B, 58(3), 481–493. |
| lme4 | 1.1-27.1 | Douglas Bates, Martin Maechler, Ben Bolker, Steve Walker (2015). Fitting Linear Mixed-Effects Models Using lme4. Journal of Statistical Software, 67(1), 1-48. doi:10.18637/jss.v067.i01. |
| nnet | 7.3-17 | Venables, W. N. & Ripley, B. D. (2002) Modern Applied Statistics with S. Fourth Edition. Springer, New York. ISBN 0-387-95457-0 |

## Analysis of results and graphics (excluding maps)

Analysis of results and graphics for the current publication was performed with R version 4.4.0 utilizing R Studio (Posit Software: RStudio 2024.04.0+735 "Chocolate Cosmos" Release (a00d0e77, 2024-04-24) for windows. <https://posit.co/products/open-source/rstudio/>). The packages employed are listed in supplementary table 3.

**Supplementary table 3:** R packages employed for analysis of results and graphics

| **package** | **version** | **citation** |
| --- | --- | --- |
|  | 4.4.0 | R Core Team (2022). R: A language and environment for statistical computing. R Foundation for Statistical Computing, Vienna, Austria.  https://www.R-project.org/ |
| collapse | 2.1.3 | Krantz, S. (2024). collapse: Advanced and Fast Statistical Computing and Data Transformation in R [Preprint]. arXiv. https://arxiv.org/abs/2403.05038  Krantz (2025). collapse: Advanced and Fast Data Transformation in R. R package version 2.1.3. doi:10.5281/zenodo.8433090. https://sebkrantz.github.io/collapse/. |
| ggplot2 | 4.0.0 | H. Wickham. ggplot2: Elegant Graphics for Data Analysis. Springer-Verlag New York, 2016. |
| patchwork | 1.3.2 | Pedersen T (2025). _patchwork: The Composer of Plots_. R package version 1.3.2, https://CRAN.R-project.org/package=patchwork. |
| rmapshaper | 0.5.0 | Teucher A, Russell K (2023). _rmapshaper: Client for 'mapshaper' for 'Geospatial' Operations_. R package version 0.5.0, <https://CRAN.R-project.org/package=rmapshaper>. |
| sf | 1.0.21 | Pebesma, E., & Bivand, R. (2023). Spatial Data Science: With Applications in R. Chapman and Hall/CRC. https://doi.org/10.1201/9780429459016  Pebesma, E., 2018. Simple Features for R: Standardized Support for Spatial Vector Data. The R Journal 10 (1), 439-446, https://doi.org/10.32614/RJ-2018-009 |
| sp | 2.2.0 | Pebesma E, Bivand R (2005). “Classes and methods for spatial data in R.” _R News_, *5*(2), 9-13. <https://CRAN.R-project.org/doc/Rnews/>.  Bivand R, Pebesma E, Gomez-Rubio V (2013). _Applied spatial data analysis with R, Second edition_. Springer, NY. <https://asdar-book.org/ |
| stringr | 1.5.2 | Wickham H (2025). _stringr: Simple, Consistent Wrappers for Common String Operations_. R package version 1.5.2, <https://CRAN.R-project.org/package=stringr> |
| tibble | 3.3.0 | Müller K, Wickham H (2025). _tibble: Simple Data Frames_. R package version 3.3.0, <https://CRAN.R-project.org/package=tibble>. |
| tidyverse | 2.0.0 | Wickham H, Averick M, Bryan J, Chang W, McGowan LD, François R, Grolemund G, Hayes A, Henry L, Hester J, Kuhn M, Pedersen TL, Miller E, Bache SM, Müller K, Ooms J, Robinson D, Seidel DP, Spinu V, Takahashi K, Vaughan D, Wilke C, Woo K, Yutani H (2019). “Welcome to the tidyverse.” _Journal of Open Source Software_, *4*(43), 1686. doi:10.21105/joss.01686 <https://doi.org/10.21105/joss.01686>. |

## Plotting of maps

Plotting of maps for the current publication was performed with R version 4.2.2 utilizing R Studio (Posit Software: RStudio 2024.04.0+735 "Chocolate Cosmos" Release (a00d0e77, 2024-04-24) for windows. <https://posit.co/products/open-source/rstudio/>). The packages employed are listed in supplementary table 4.

**Supplementary table 4:** R packages employed for plotting of maps

| **package** | **version** | **citation** |
| --- | --- | --- |
|  | 4.2.2 | R Core Team (2022). R: A language and environment for statistical computing. R Foundation for Statistical Computing, Vienna, Austria.  https://www.R-project.org/ |
| sf | 1.0-21 | Pebesma, E., & Bivand, R. (2023). Spatial Data Science: With Applications in R. Chapman and Hall/CRC. https://doi.org/10.1201/9780429459016  Pebesma, E., 2018. Simple Features for R: Standardized Support for Spatial Vector Data. The R Journal 10 (1), 439-446, https://doi.org/10.32614/RJ-2018-009 |
| sp | 2.2-0 | Pebesma E, Bivand R (2005). “Classes and methods for spatial data in R.” R News, *5*(2), 9-13. https://CRAN.R-project.org/doc/Rnews/.  Bivand R, Pebesma E, Gomez-Rubio V (2013). Applied spatial data analysis with R, Second edition. Springer, NY. https://asdar-book.org/. |
| tibble | 3.3.0 | Müller K, Wickham H (2025). tibble: Simple Data Frames. R package version 3.3.0, https://CRAN.R-project.org/package=tibble |
